# Candidate genes associated with heat stress and breeding strategies to relieve its effects in dairy cattle: a deeper insight into the genetic architecture and immune response to heat stress

**DOI:** 10.3389/fvets.2023.1151241

**Published:** 2023-09-13

**Authors:** Destaw Worku, Jamal Hussen, Giovanna De Matteis, Benjamin Schusser, Mohanned Naif Alhussien

**Affiliations:** ^1^Department of Animal Science, College of Agriculture, Food and Climate Sciences, Injibara University, Injibara, Ethiopia; ^2^Department of Microbiology, College of Veterinary Medicine, King Faisal University, Al-Ahsa, Saudi Arabia; ^3^Council for Agricultural Research and Economics, CREA Research Centre for Animal Production and Aquaculture, Monterotondo, Rome, Italy; ^4^Reproductive Biotechnology, TUM School of Life Sciences, Technical University of Munich, Freising, Germany

**Keywords:** heat stress, dairy cattle, genomic regions, candidate genes, immune response, heat tolerance

## Abstract

The need for food products of animal origin is increasing worldwide. Satisfying these needs in a way that has minimal impact on the environment requires cutting-edge technologies and techniques to enhance the genetic quality of cattle. Heat stress (HS), in particular, is affecting dairy cattle with increasing frequency and severity. As future climatic challenges become more evident, identifying dairy cows that are more tolerant to HS will be important for breeding dairy herds that are better adapted to future environmental conditions and for supporting the sustainability of dairy farming. While research into the genetics of HS in the context of the effect of global warming on dairy cattle is gaining momentum, the specific genomic regions involved in heat tolerance are still not well documented. Advances in omics information, QTL mapping, transcriptome profiling and genome-wide association studies (GWAS) have identified genomic regions and variants associated with tolerance to HS. Such studies could provide deeper insights into the genetic basis for response to HS and make an important contribution to future breeding for heat tolerance, which will help to offset the adverse effects of HS in dairy cattle. Overall, there is a great interest in identifying candidate genes and the proportion of genetic variation associated with heat tolerance in dairy cattle, and this area of research is currently very active worldwide. This review provides comprehensive information pertaining to some of the notable recent studies on the genetic architecture of HS in dairy cattle, with particular emphasis on the identified candidate genes associated with heat tolerance in dairy cattle. Since effective breeding programs require optimal knowledge of the impaired immunity and associated health complications caused by HS, the underlying mechanisms by which HS modulates the immune response and renders animals susceptible to various health disorders are explained. In addition, future breeding strategies to relieve HS in dairy cattle and improve their welfare while maintaining milk production are discussed.

## 1. Introduction

In many regions of the world, climate change is likely to lead to higher average temperatures, humidity, hotter daily maximum and more frequent heat waves, which could increase HS for livestock. The cattle population contributes to and is affected by the projected increase in global warming. As global demand for food products of animal origin is expected to rise by 70% by 2050, this demand must be met in a manner that has a negligible impact on the environment by improving the genetic quality of livestock using cutting-edge technologies and techniques (1). Temperature and humidity levels that exceed certain comfort zones lead to worsening the environmental conditions for dairy cattle and sub-tropical areas. Thus, dairy cows are more prone to environmental HS because of the rigorous selection for high milk yield combined with the high metabolic heat produced from the fermentation of additional dry matter during lactation (2, 3). As the high-producing dairy cows experience HS which negatively affects milk production, fertility, health and welfare traits (4–9), there is an urgent need to mitigate the effects of HS on dairy cows to improve the profitability and sustainability of the dairy production system.

The impact of HS in the dairy industry can be mitigated through a combination of different approaches including physical adjustments of the environment, enhanced nutrition, and management practices (10, 11). However, these alternatives are usually not cost-effective and tend to be unfeasible in the long term, especially in extensive or semi-intensive production systems (12). Additionally, these strategies might not work well in pasture-based dairy production systems where cows are exposed to solar radiation for a large part of their time while grazing (13). One strategy to permanently resolve the problem is genetic selection for improved heat tolerance combined with better management practices. It is more important to consider that the success of genetic selection of economically important traits depends on its heritability and additive genetic variation. However, physiological indicators of HS traits, including respiration rate (RR), rectal temperature (RT), and drooling score (DS) are low heritability traits (12, 14, 15) governed by a large number of quantitative trait loci (QTL), each with a small effect, so the possibility of rapid genetic progress through traditional genetic selection is low. Therefore, a better understanding of the genetic architecture of a trait, encompassing the underlying candidate causal variants with their corresponding effects and the trait variation that can be explained by genetic variation related to HS would be essential to derive genomic prediction and achieve rapid genetic progress for heat tolerance trait in dairy cattle. This prompts the need to identify genomic variants associated with heat tolerance phenotype, thus contributing an additional feature in the headway of genomic selection (GS) of heat-resistant dairy animals (16).

Recent developments in high-throughput sequencing techniques and emerging genomic data have opened up the opportunity to decipher the genomic variants affecting economically important traits in cattle. For a handful of economic traits, QTL mapping, transcriptome profiling and GWAS were conducted under HS conditions to gain better insight into the genetic basis for HS response in cattle. Several recent GWAS and transcriptome studies have been conducted to uncover candidate genes and causal variants associated with various indicators of heat tolerance traits in a wide range of dairy cattle breeds (11, 12, 14, 15, 17–24). In addition to GWAS, selection signatures can be used to associate candidate genes or variants subject to selection with traits of interest that can subsequently be used in GS (25). There are significant distinctive genetic traces or prints left behind in the genome that were subjected to selection called selection signature (26). Recently, genomic data provided additional possibilities to identify these footprints or genomic areas of selection linked to adaptation and productivity in cattle (27, 28). There has been an upsurge interest in identifying and exploring these genomic signatures of selection using high-density SNPs and revealed candidate genomic regions associated with environmental adaptation and thermotolerance in cattle (29–31). Since genome-wide significant single nucleotide polymorphism become affordable, GS has been tested and recognized by the dairy industry as a successful and easy-to-implement alternative (1). Nevertheless, a comprehensive review of the genetic basis of HS and milk production response in dairy cattle is scarce in the literature. Therefore, this review set out to undertake a comprehensive search for candidate genomic regions and/or genes and selection signatures associated with HS response in dairy cattle with the potential for simultaneous improvement of heat resistant and milk yield traits in dairy cows. Because of its importance for future breeding programs, the main mechanisms by which thermal stress affects essential immune system functions and influences animal health and welfare are also discussed. In addition, breeding strategies and recent approaches to mitigate HS in dairy cows were also discussed in this review.

## 2. Heat stress, milk production, and genetic basis in dairy cattle

The effects of HS on dairy cows are profound, and contribute significantly to lower overall milk production, fertility, and impaired health and welfare (4–8). In particular, high-yielding dairy cows are more susceptible to the stressful effects of HS (13). It is noteworthy that continuous selection for high milk yield in dairy cows, combined with the increased metabolic heat produced by the fermentation of extra dry matter during lactation (2, 3), compromises the maintenance of homeothermy under HS conditions. For instance, when lactating dairy cows are exposed to high ambient temperature and high relative humidity for prolonged periods, their ability to dissipate heat gained through metabolic process and from the environment is reduced, making them susceptible to HS (32, 33). To reduce their heat load, the cow reduces her feed intake and consequently milk production (34). Despite the fact that lactating cows are more susceptible to heat stress, HS exposure during the dry period affects subsequent lactation and has long-lasting effects on the progeny (35, 36). In order to assess the effects of HS in lactating and dry cows, particularly the simultaneous effect of temperature and relative humidity, the temperature-humidity index (THI) is widely used (34). On the other hand, changes in production traits, especially milk yield, have often been used to determine the THI threshold for HS (6). It was worth mentioning that, increasing THI linearly decreases milk production in lactating dairy cows (6, 37, 38). In terms of losses, HS causes production losses of 600–900 kg of milk per cow/lactation (39). Routine selection of dairy cows for higher milk yield may result in poor response of dairy cows to HS, due to the antagonistic genetic relationship between production level and specific ability to respond to HS (11, 37, 40). This clearly indicates the urgent need to develop sustainable strategies to reduce the detrimental effects of HS on milk yield in dairy cattle.

Although HS affects milk yield in dairy cows, more attention should be paid to how HS affects milk composition. For example, previous studies reported a significant correlation between increasing somatic cell count (SCC) in milk and increasing THI in dairy cattle (41–43). In the context of HS, consideration of the environmental sensitivity of fatty acids in milk for genome analysis and gene annotation is a novel approach, where HS exacerbates changes in milk fatty acid profiles including stearic acid (C18:0), polyunsaturated fatty acids (PUFA), saturated fatty acids (SFA) and unsaturated fatty acids (UFA) during early lactation in high-yielding cows (18, 44). It is argued that exposure to HS in a high THI environment appears to inhibit milk fat and protein synthesis in lactating dairy cows, which in turn directly affects the synthesis of energy-corrected milk yields (45). More fundamentally, heat exposure appears to impair the capacity of the mammary gland to synthesize milk proteins by down-regulating the expression of key milk protein genes such as *β-casein* and *butyrophilin* (46). Additionally, HS negatively affects the synthesis of milk proteins by decreasing the transcription of metabolism-related genes and increasing inflammation-related genes (47). Heat stress could also alter the triacylglycerol profile of milk, which is characterized by a decrease in triacylglycerol groups with predominantly short-chain FAs to medium-chain FAs and a concomitant increase in groups with predominantly long-chain FAs (48). In addition, the authors demonstrated that HS significantly decreases the content of some polar lipid classes, especially lysophosphatidylcholine, which appears to be a lipid marker of HS in dairy cows.

Genetic factors in cattle have been recognized as a main feature that can explain part of the differences in response to HS in dairy cows. For example, Otto et al. (15) performed a GWAS in Gir × Holstein F2 experimental population to identify genetic markers responsible for genetic variation in response to HS and used the breed of origin of alleles (BOA) approach to evaluate the origin of marker alleles of candidate genes. The authors found that most animals that responded better to the effects of HS had 2 alleles of the Holstein breed, while a high proportion of heat-stressed animals had 2 alleles of the Gir breed. This indicates Holstein breed alleles could be related to a more complex response to HS effects, which could be explained by the fact that Holstein animals are more affected by HS than Gir animals, and thus having more complex genetic mechanisms to defend the body from the harmful effects of HS. Therefore, revealing the origin of marker alleles of candidate genes for heat tolerance in dairy cattle populations can help in understanding the genetic variation of the trait, which is subsequently used to estimate breed-specific SNP effects to improve genomic prediction for heat tolerance and production traits in dairy cattle.

Most economically important traits in farm animals are associated with polygenes located at QTLs that are widely distributed throughout the genome. With the availability of high-throughput sequencing technologies, detection of a number of high-impact QTL in dairy is possible, suggesting genomic regions and genes responsible for significant differences in the yield and composition under HS conditions. In addition, a wide range of genomic approaches have been employed to identify genetic variants linked to physiological indicators of response to HS. However, the heat tolerance trait is highly polygenic and influenced by multiple variants, each with small effects on the phenotype, suggesting that the trait may be more suitable for genomic selection tools such as those currently used in the Australian dairy industry (49, 50), than for methods that exploiting few QTLs with major effects. In QTLs, it is difficult to find a few markers that explain a large proportion of genetic variance, but when found, they can revolutionize the selection process of dairy cows tolerant to HS. For example, the slick-hair gene is clearly linked to heat tolerance in dairy cows as a result of a single gene effect (51), i.e., improved ability to dissipate heat (52). Moreover, the integration of omics information could help to reveal nodes of the HS control network and eventually find a panel of markers that can be used in the selection of heat-tolerant dairy animals with higher productivity. Overall, heat tolerance in dairy cows is a complicated phenomenon that calls for the combination of phenotypes and omics information to provide accurate tools for selective breeding without compromising productivity.

## 3. Impact of heat stress on immune response in dairy cattle

**Figure 1:**
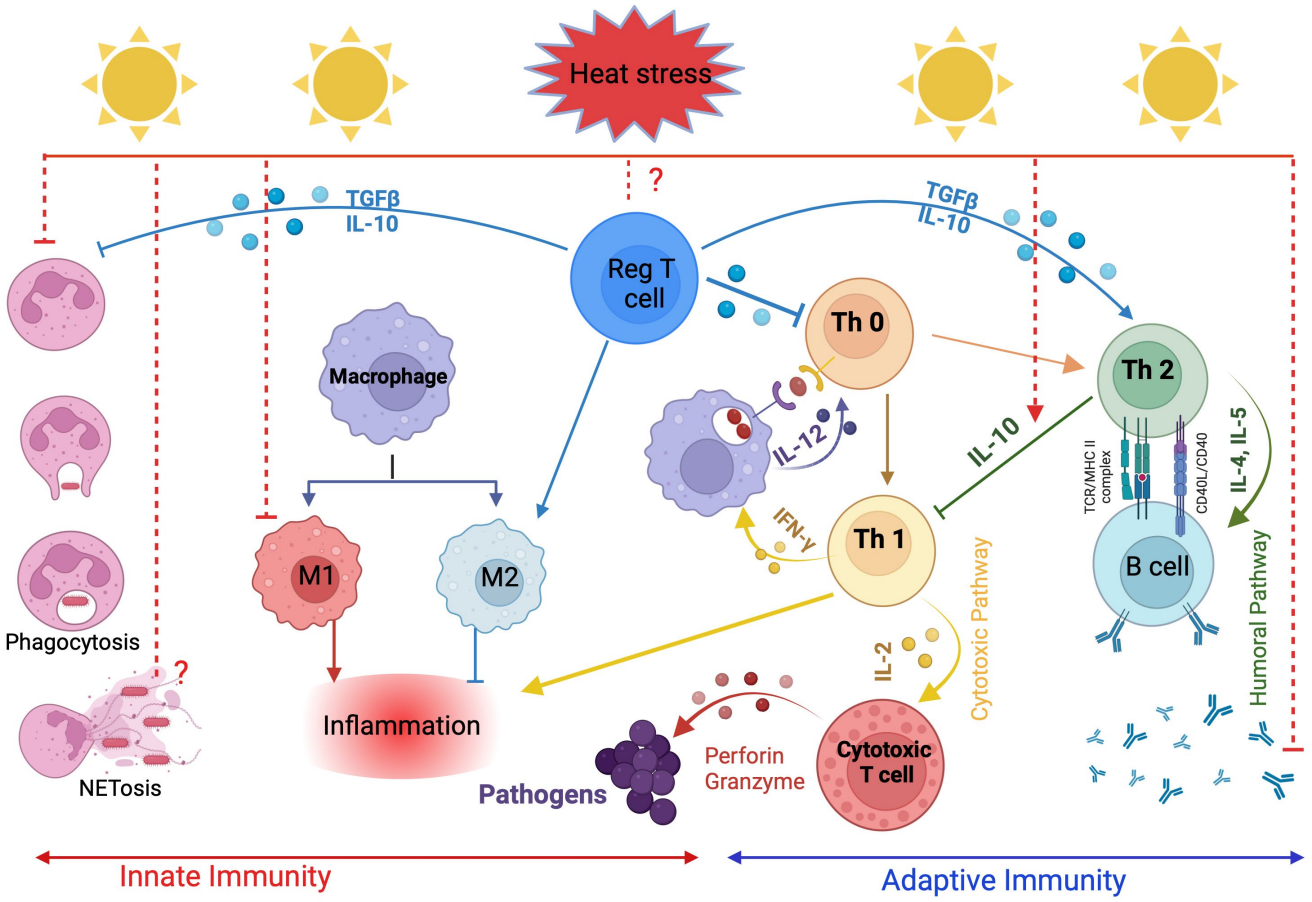
Effects of heat stress on the innate and adaptive immune response in cattle. Created with BioRender.com.

Since HS suppresses the immune and endocrine system, exacerbate health and metabolic disorders (53, 54) and ultimately affect the performance of dairy cattle, understanding the underlying mechanism by which HS modulates the immune response of cows is crucial and must be considered in future breeding strategies. It is important to remember that, Lengi et al. (55) observed significantly lower concentrations of granulocytes in milk from heat-stressed dairy cows. Neutrophils, the most common type of granulocyte found in SCCs, are recruited by chemo-attractants to infection sites such as interleukin-8 (*IL-8*), including the mammary gland, where they phagocytose and destroy pathogens (56). During HS, the mammary gland’s ability to respond to infection is compromised, as evidenced by a decline in the concentration of viable granulocytes there. Heat stress possesses a significant negative impact on both arms of the immune system, the innate and the acquired immunity. Exposure to HS causes a variety of changes in the physiology of the body, including activation of the hypothalamic–pituitary–adrenal (HPA) axis and secretion of glucocorticoids (7, 57). Immune cells have glucocorticoid receptors which allow glucocorticoids to stimulate immune response during acute stress, however, the functions of the immune system get impaired when the exposure to stress is elongated (chronic stress) (58). The major immune cells that contribute to innate immunity are phagocytes (i.e., neutrophils, monocytes, macrophages), cytokines and inflammatory mediators-producing cells (i.e., neutrophils, macrophages, mast cells, natural killer cells). The acquired (adaptive) immune response is composed of T cells and B cells that contribute to cell-mediated immunity and humoral immunity, respectively (59, 60). Although the innate and acquired immune cells are distinct from each other, their interplay is evident through their specific roles in initiating and regulating immune responses (Figure 1).

Of the cells of the innate immune system, neutrophils are the first line of defense, and they kill and eliminate invading microorganisms by phagocytosis, degranulation, and release of neutrophil extracellular traps (61). Heat stress impairs the potential of bovine neutrophils for phagocytosis and reactive oxygen species (ROS) production under *in vitro* and *in vivo* conditions (41, 53, 62). This indicates that HS impaired the pathogen recognition ability of neutrophils and suppressed inflammatory immune response to pathogens. Mononuclear phagocytes, including blood monocytes and their derived tissue macrophages, are another important player in the innate defense against invading pathogens. They are divided into functional subsets based on their roles during the inflammatory response. Classically activated M1 monocytes/macrophages are pro-inflammatory cells with a higher potential for antimicrobial functions, while alternatively activated M2 monocytes/macrophages have an anti-inflammatory phenotype with a special role during the resolution of inflammation (63, 64). Catozzi et al. (65) exposed bovine monocytes to high temperatures and investigated the expression profile of genes responsible for monocyte/macrophage polarization. They reported a polarization of monocytes from a classical M1 to a non-classical M2 phenotype which is associated with suppressed innate immune response and could favor the humoral immune response. An M2-like macrophage phenotype, which was identified by the high expression of the M2 cell marker *CD163*, was also found in the intestinal lymphoid tissue of dairy cows under HS (66). Antigen presentation is an important immune process to translate information obtained by antigen-presenting cells (APCs) into specific actions of the desired T-cell subsets. Although there is scanty information on the effect of thermal stress on antigen processing and presentation in bovines, several studies on humans and mice reported that heat shock impairs the presentation of exogenous antigens by *MHC II* and suppresses co-stimulatory functions in APCs such as macrophages, dendritic cells, or B cells (67).

The desired cellular or humoral immune responses are triggered based on a repertoire of pro and anti-inflammatory cytokines mainly produced by CD4+ T helper cells (68). Polarized T helper 1 cells (Th1) activate cellular immunity and enhance antimicrobial responses against intracellular pathogens by secreting type 1 pro-inflammatory cytokines such as tumor necrosis factor-α (*TNF-α*), interferon-γ (*IFN-γ*) and interleukin-12 (*IL-12*). Polarized T helper cells (Th2), on the other hand, secrete type 2 cytokines (like *IL-4*, *IL-5, IL-10* and *TGF-β*) that play a regulatory role during the immune response (*IL-10* and *TGF- TGF-β*) favorite allergic reactions (*IL-4* and *IL-5*), and stimulate B cells for the production of special antibody isotypes (*IL-4* and *IL-13*) to fight extracellular bacteria and helminths (69). Heat stress stimulates the secretion of stress hormones, the expression of heat shock proteins (*HSPs*) and alters the cytokine profile, which in turn alters the Th1:Th2 balance. Several studies have shown that thermal stress suppresses the secretion of Th1 cytokines (cellular immunity) and stimulates the secretion of Th2 cytokines (humoral immunity), which impairs cell-mediated immunity, decreases lymphocyte proliferation, and increases the susceptibility of heat-stressed animals to various infectious diseases (7, 54). However, the switch to Th2 cytokines does not ensure an adequate humoral immune response (Figure 1). On the contrary, *IgG* response to an innocuous antigen and to booster immunizations was significantly lower in heat-stressed compared to cooled cows during the dry period and after parturition (53). This is also confirmed by the reduced number of *CD21*-positive B cells in cows exposed to HS compared to normal cows (70). In addition, Hu et al. (71) reported that chronic HS significantly impairs the immune response to foot and mouth disease (FMD) vaccination by decreasing *IgG2a* levels and suppressing cytotoxic T-cell response, T-cell proliferation and *IFN-γ* expression in both *CD4+* and *CD8+* cells.

Regulatory T cells (Tregs) secrete anti-inflammatory cytokines such as *TGF-β* and *IL-10*, which maintain host immune homeostasis by balancing Th1:Th2 activity and are characterized by the expression of the *IL-2* receptor *CD25* and the transcription factor forkhead box P3 (*FOXP3*) (72). Although the effects of HS on Tregs in cattle are unknown, studies in mice have shown that the number of Tregs decreases and their immunosuppressive capacity is impaired in response to HS exposure (73, 74). Therefore, maintaining the optimal function of Tregs to balance the activity of inflammatory cytokines and the Th1:Th2 ratio is essential for maintaining homeostasis and preventing inflammatory diseases in cattle under HS conditions. The mechanism by which HS modulates the immune response and renders dairy cows susceptible to invading pathogens explains the higher incidence of mastitis, metritis, respiratory disease, displaced abomasum, retained foetal membranes and lameness during HS (5). Of interest, Alhussien and Dang (41) have reported genetic variability in resilience to HS in Indian native breeds of tropical environments. The mammary defense mechanism and milk production were less affected in Gir and Tharparkar cows as compared to Sahiwal cows under HS conditions (41). This highlights that there is variation in heat resilience among Zebu breeds indigenous to warm climates that should be considered in breeding programs. Especially during the late-gestation and prepartum period, HS of the dairy cow not only impaired their post-partial immune function resulting in higher susceptibility to uterine infections (75) but also carried over negative effects on the passive transfer of colostral *IgG* to the newborn dairy calves and compromised their immune function (76).

## 4. Genetic evaluation of heat tolerance in dairy cattle

It is evident that substantial genetic variation that explains part of the variation in response to heat tolerance exists among animals (52, 77). One way to determine the nature and extent of genetic variation for heat tolerance is to genetically assess HS indicator traits in dairy cattle. Numerous research on genetic evaluation of heat tolerance in dairy cattle has been based on analysis of performances under HS conditions. With substantial advancement in the area of quantitative genetics, phenomics and genomics, studies on genetic and genomic evaluation expanded to a wide range of HS indicator traits that optimally includes several other indicators of thermoregulation targeted for heat tolerance improvement in dairy cattle breeds (14, 18, 44, 50). Besides indicator traits for HS, progress has also been made in the development of resilience indicators traits for dairy cattle breeding (78–81), which encompasses multiple traits and could be considered in the breeding goals to accelerate genetic progress and enhance farm profitability (2).

**Figure 2:**
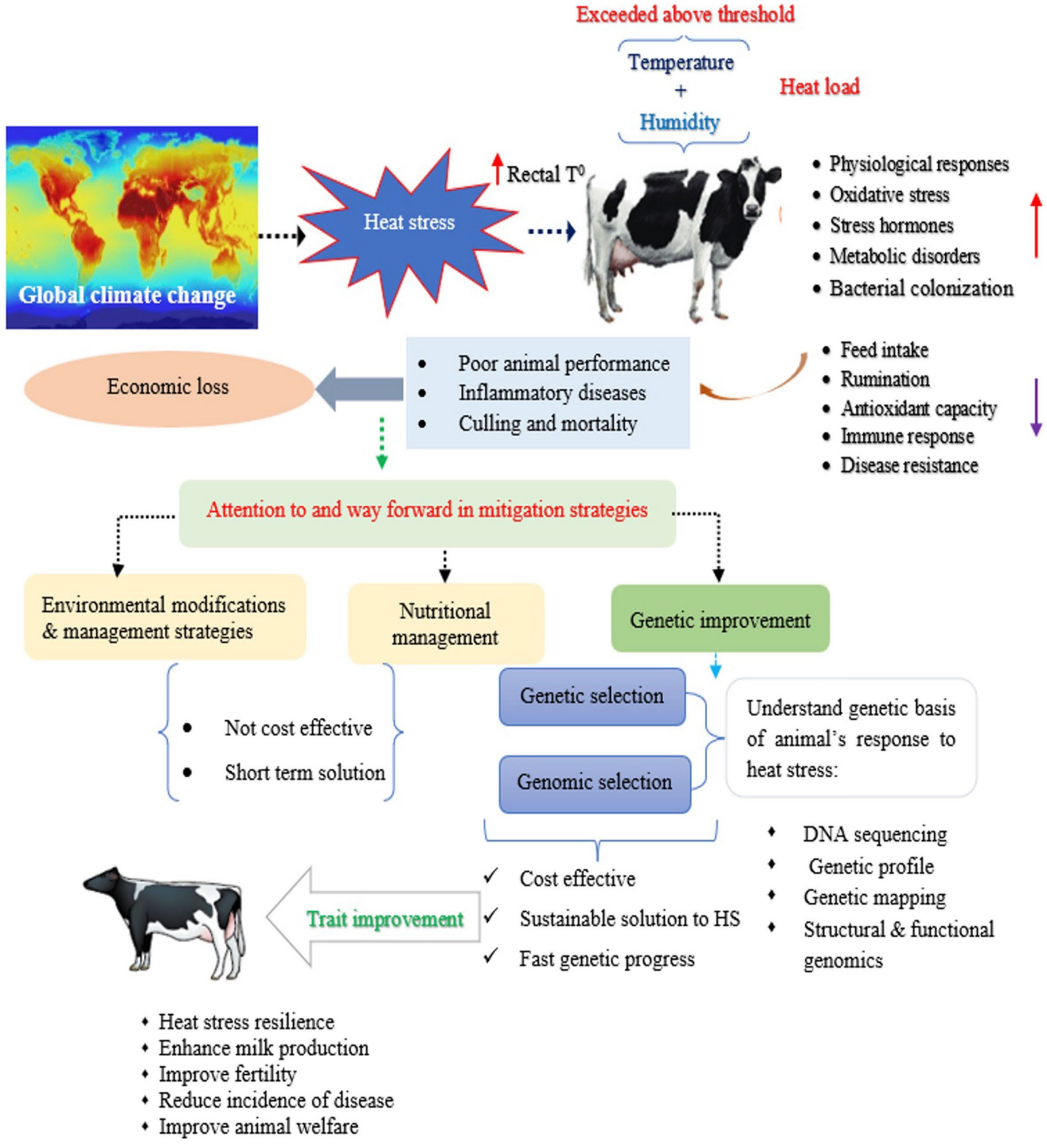
An overview of heat stress and its cumulative effect on dairy cattle, and mitigation strategies.

In an attempt to evaluate the genetic effect of HS and genetic tolerance for milk production and its components in dairy cattle, genetic parameters were estimated for several indicators of HS phenotype and milk production response as well as resilience indicators traits in dairy cattle (12, 50, 79, 80, 82–86). Alternatively, several HS indicators, for example, RT, RR, and DS are common physiological indicators used to identify heat-tolerant animals within a breed (13, 87). Another way to assess heat tolerance is to monitor changes in milk production traits under warm environmental conditions (88–91). Therefore, these HS indicator traits are often considered when aiming to genetically evaluate HS response in animals. In this context, the heritability estimates for RT, RR, and DS are low, with heritability estimates ranging from 0.03 (DS) to 0.06 (RT) in the Holstein-Friesian cattle population (14). However, Dikmen et al. (87) reported a higher estimate of heritability (0.17) for RT in the Holstein cattle population. Considering an average THI threshold of 62 for milk production traits, heritability estimates increased with increased THI above its threshold of 62 for milk yield (0.20–0.23) and protein yield (0.14–0.16) and remained firm for fat yield (0.17) in Holstein cattle (83). With a THI of 76, Sungkhapreecha et al. (89) reported heritability estimates varied from moderate to low, with values of 0.344 for milk yield, 0.087 for somatic cell score and 0.061 for milk fat to protein ration in tropical dairy cattle breeds. A reduction in additive genetic variance in milk yield was observed with increasing THI levels in purebred Zebu cattle (90). For milk fatty acid profiles, heritability estimates were higher for UFA (UFA, MUFA, and PUFA), while lower for SFA traits (SFA, C16:0, and C18:0) under the THI of 10 degrees over its threshold of 68 than an average THI threshold of 68 in Brazilian Holstein cattle (82). Similar findings by Hammami et al. (91) observed higher heritability estimates for PUFA and C18:1 at high THI levels in Belgium Holstein cows. In addition, greater additive genetic variances were estimated for C18:0, PUFA, and UFA uder HS conditions compared to thermo- neutral conditions during the first lactation period, reflecting environmental sensitivity during early lactation in high-yielding Holstein dairy cows (18). This genetic variability for milk fatty acid profiles in relation to THI impedes motivation to consider these traits in the breeding objective for genetic selection to leverage heat tolerance in dairy cattle, thus, response to selection would be expected (Figure 2). Regardless of the effects of the lactation stage, Bohlouli et al. (44) estimated the greatest additive genetic variances for C18:0, MUFA, PUFA, and UFA at high THI than under temperate climate conditions, whereas fat yield, palmitic acid (C16:0), and SFA decreased with increasing THI. Nevertheless, Bohlouli et al. (44) identified C18:0, MUFA, PUFA, and UFA as climate-sensitive traits due to large genetic variance at the extreme ends of the THI scale, indicating the future possibility of improving thermotolerance in dairy cattle through genetic selection. Most importantly, larger genetic variation was observed for C18:1, indicating the greatest sensitivity to HS conditions in a tropical climate (91). With respect to genetic trends, the genetic components of HS have had negative effects on milk production and quality traits of dairy cattle over the years (37, 90). Despite the low heritability estimates for physiological indicator traits, incorporation of these traits in the genetic evaluation programs and consider these traits in the selection index helps to achieve optimum genetic progress for heat tolerance, while maintaining milk production traits in dairy cattle.

## 5. Genomic regions and candidate genes associated with heat tolerance in dairy cattle

The ability of an animal to respond to HS can be assessed by physiological indicators of HS traits, such as RT, RR, and DS, which increase when the animal is exposed to a warm environment (13, 87). Another way to assess heat tolerance is to monitor changes in milk production traits or analyze milk yield trait alternations by HS (45, 88). These physiological indicators of HS traits shown to be heritable (12, 14, 15, 49, 87), indicate that genetic gain can be made through selection. Notably, the GxE work by Cheruiyot et al. (77) reported the existence of substantial genetic variation for heat tolerance in dairy cattle, which can provide a better insight into the genetic selection of thermo-tolerant dairy cows. However, heat tolerance is a complex trait, governed by a myriad of adaptative responses (behavioral, physiological, cellular, etc.) that tend to be polygenic, and as a result, many genetic variants, each with small effects contribute to the phenotype. In these cases, the heat tolerance trait has been associated with a number of QTL regions and candidate genes (14, 15, 18, 92). This polygenicity of a trait can be a challenge in achieving rapid genetic progress for heat tolerance in dairy cattle, particularly in cases when the heritability of a trait is low. To avoid these problems, genome-wide DNA markers should be used to capture the effects of many genetic variants. In this regard, the application of genomic selection is ideal and is increasingly used to accelerate genetic progress, by increasing the accuracy of selection and shorting generation intervals.

Identifying genome-wide markers and pathways underpinning the genetic mechanisms that increase thermotolerance in dairy cattle is at the infancy stage, but it is gaining momentum considering the increasing challenges imposed by climate change, as shown by the recent increase in published studies (2, 11, 12, 14, 18, 92–96). Genome-wide association studies are most commonly used to test tens if not hundreds of thousands of genomic variants to find those statistically associated with heat tolerance traits in cattle. Although GWAS have led to the identification of trait-associated genomic variants, the researchers have conducted validation of GWAS to prioritize the variants most likely to be causal to thermo-tolerance and to provide several biological insights that can be leveraged to minimize HS while maintaining milk production in dairy cattle using a large dataset (2, 12, 14, 93). As such, biological validation of candidate genes for RT as HS indicator in Holstein cattle observed that QTL regions on BTA3, 4, 8, 13, 14, 17 and 29 are perhaps the most significant regional association signals with heat tolerance in dairy cattle (12). A similar study by Luo et al. (14) revealed that the genomic regions on BTA3, 6, 8, 12, 14, 21 and 24 were significantly associated with HS response in Holstein dairy cows. Several other previous studies confirmed the significant effects of QTL regions on BTA6 and 17 for RT during HS in Holstein cattle (15, 16). Luo et al. (94) reported the presence of a genomic region on BTA14 strongly associated with thermotolerance in Australian Holstein cattle as part of a conditional GWAS strategy. Of interest, Sigdel et al. (11) performed whole-genome mapping to identify candidate genes associated with milk production traits under HS conditions in US Holstein cows and demonstrated a significant association between BTA5, 14, and 15 and milk production. Interestingly, Bohlouli et al. (18) claimed that the highest number of significant SNP markers on BTA14 were significantly associated with HS response of milk fatty acids profiles (e.g., PUFA) in the first and third lactation stages under thermoneutral and HS conditions in dairy cows. This provided stronger evidence, suggesting that QTL effects in BTA14 altered the concurrent improvement of milk production and heat tolerance traits in dairy cows. Previously, Dikmen et al. (24) identified QTL regions on BTA4, 5, 16, 24, and 26 correlated with rectal temperature for heat-stressed Holstein animals using the one-step genomic BLUP approach. Similarly, regions significantly affecting RT in lactating Holstein cows were detected in BTA4, 6, and 24 (16). In addition, the region on BTA22 (95) and BTA26 (96) was reported to have a significant association with heat tolerance in dairy cows. According to the results of the literature search, the candidate genomic regions associated with heat tolerance traits in dairy cows are located on BTA3, 5, 14, and 16, respectively. Despite the fact that BTA14 carries important genes for traits of economic importance, the relationship between BTA14 and milk-related traits is well described in the literature. For example, the significant SNP markers on BTA14 located within or near the potential candidate genes *DGAT1*, *PPP1R16A*, *FOXH1*, and *PLEC* had the greatest effects on fatty acid across lactations under different conditions of HS in dairy cows (18). Therefore, the specific candidate genes on BTA3, BTA5, and BTA14 may hold promise for simultaneously improving heat tolerance and milk-related traits in dairy cattle. Overall, these QTL regions shed new light on candidate genes that are potentially associated with heat tolerance trait architecture, while maintaining milk production traits in dairy cattle. In summary, with the emerging genomic data, a growing list of candidate genes is being uncovered for genetic improvement to enhance aspects of heat tolerance, while maintaining milk production traits in dairy cattle (Table 1).

**Table 1:** Candidate genes associated with various heat tolerance traits in dairy cattle population.

BTA	Candidate genes identified	Approaches	Trait	Breed	No. samples/animals	Country	References
1	*SOD1*	GWSS	TR	*B. indicus*	48	Africa	(95)
2	*DNAJC8* ^*^	GWSS	HT	EASZ	92	Kenya	(97)
		GWSS	HT	EASZ	425	Kenya	(98)
	*OLA1*	WGSS	HT	Muturu	10	Nigeria	(99)
	*NDUFB3* and *DIS3L2*	GWSS	Adaptation	HF	1,092	China	(100)
	*SERPINE2*	SNP	HSR	HF	455	US	(16)
3	*SPAG17* ^*^	WssGWAS	HSR	HF	3,200	China	(14)
		GWAS	RT	HF	1,114	China	(12)
	*CMPK1*	GWSS	HT	Zebu	275	Eth and Bang	(31)
	*PRLH*	GWSS	TR	*B. indicus*	48	Africa	(95)
	*MLPH* and *RAB17*	WGSS	TR	African cattle	48	Africa	(101)
	*DNAJB4*	GWSS	HT	Ongole	20	India	(102)
	*HSD17B7*	SNP	RR	HF	450	US	(16)
4	*KBTBD2* and *LSM5*	ssGBLUP	RT	HF	1,451	US	(24)
	*GRM8*	GWAS	HT	HF	300	Mexico	(92)
	*ASIC3*	GWSS	HSR	African cattle	278	North Africa	(103)
	*CFTR*	WGSS	TR	African cattle	48	Africa	(101)
	*AQP1*	GWSS	Adaptation	HF	1,092	China	(100)
5	*CDKN1B*	GWAS	TT	HF	17,522	US	(11)
	*MCAT*	GWAS	HT	HF	423	Italy	(96)
	*SLCO1C1* and *PDE3A*	ssGBLUP	RT	HF	1,451	US	(24)
	*FKBP4*	GWSS	HT	GIR	24	India	(102)
	*DNAJC14*	GWSS	HT	EASZ	425	Kenya	(98)
	*HOXC12, HOXC13* and *ITPR2*	WGSS	TR	African cattle	48	Africa	(101)
6	*TMEM33*	WssGWAS	HSR	HF	3,200	China	(14)
	*NPFFR2*	GWAS	HT	HF	29,107	Australia	(93)
	*GRXCR1*	GWSS	HT	THA	24	India	(102)
7	*HSPA4*	GWSS	HT	Zebu	275	Eth and Bang.	(31)
	*HSPA9* ^*^	GWSS	HT	*B. indicus*	48	Africa	(95)
		GWSS	HT	EASZ	92	Kenya	(97)
	*DNAJC18* ^*^	GWSS	HT	EASZ	92	Kenya	(97)
		GWSS	HT	EASZ	425	Kenya	(98)
8	*TLR4*	GWAS	HT	HF	300	Mexico	(92)
	*IL6*	GWSS	HT	ONG	20	India	(102)
	*RGS3*	GWSS	Adaptation	HF	1,092	China	(100)
9	*RTN4IP1*	WssGWAS	RR	HF	3,200	China	(14)
	*SGK1*	WGSS	TR	African cattle	48	Africa	(101)
10	*RBM25*	Transcriptome	HSR	HF	36	China	(17)
	*SMAD3*	GWAS	HT	HF	300	Mexico	(92)
	*SPTLC2*	GWSS	Adaptation	HF	1,092	China	(100)
11	*ENSBTAG00000048091, PAEP* and *EPPK1*	GWAS	UFA	HF	3,777	Germany	(18)
	*SLC9A4*	WGSS	TR	African Cattle	48	Africa	(101)
12	*HSPH1*	GWSS	HSR	African Cattle	278	North Africa	(103)
	*INTS6*	WGSS	HT	Muturu	10	Nigeria	(99)
13	*FAM107B*	GWAS	RT	HF	1,114	China	(12)
14	*TSNARE1, RALYL*	GWAS	RT	HF	1, 114	China	(12)
	*HSF1* ^*^	GWAS	HT	HF	29,107	Australia	(93)
		GWAS	TT	HF	17,522	US	(11)
		GWAS	HT	HF	423	Italy	(96)
		GWSS	HT	Zebu	275	Eth and Bang.	(31)
15	*GRIA4*	GWAS	HT	HF	29, 107	Australia	(93)
	*MAPK8IP1*	GWAS	TT	HF	17, 522	US	(11)
	*PGR*	SNP	RT	HF	435	US	(16)
16	*CALCR* and *GHR*	GWAS	HT	HF	2,907	Australia	(93)
	*SNORA19, RFWD2, SCARNA3, CEP170* and *PLD5*	ssGBLUP	RT	HF	1,451	US	(24)
	*MTOR*	GWSS	HT	Zebu	275	Eth and Bang.	(31)
	*SCNN1D*	WGSS	TR	African cattle	48	Africa	(101)
	*PEX14*	GWSS	Adaptation	HF	1,092	China	(100)
17	*GATB*	WssGWAS	RR	HF	3,200	China	(14)
	*LIF, OSM, TXNRD2* and *DGCR8*	GWAS	∆RT	Gir X HF	341	Brazil	(15)
18	*MVD*	GWSS	HSR	African cattle	278	North Africa	(103)
19	*LUC7L3*	Transcriptome	HSR	HF	36	China	(17)
	*DNAJC7*	GWSS	HT	EASZ	425	Kenya	(98)
	*RAB37*	WGSS	TR	African cattle	48	Africa	(101)
	*KRT24*, *KRT25, KRT26, KRT27, KRT28* and *HSPB9*	GWSS	Adaptation	AFR, DRA, NGI	90	South Africa	(104)
	*HSPB9* ^*^	GWSS	HT	EASZ	425	Kenya	(97, 98)
20	*SLC45A2*	WGSS	TR	African cattle	48	Africa	(101)
21	*BTBD7*	WssGWAS	DS	HF	3,200	China	(14)
	*PTPN9*	GWSS	Adaptation	HF	1,092	China	(100)
23	*HSPA1L* and *HSPA1B*	GWSS	HT	THA and GIR	24	India	(102)
	*UBD*	GWSS	Adaptation	HF	1,092	China	(100)
24	*PMAIP1*	WssGWAS	RT	HF	3,200	China	(14)
	*NCAD*	ssGBLUP	RT	HF	1,451	US	(24)
	*MC5R*	WGSS	TR	African cattle	48	Africa	(101)
25	*SBK1*	WssGWAS	RT	HF	3,200	China	(14)
26	*HSPA12A*	GWSS	HT	ONG	20	India	(102)
	*GOT1*	ssGBLUP	RT	HF	1,451	US	(24)
29	*CHORDC1*	WssGWAS	RR	HF	3,200	China	(14)
	*PHRF1*	GWAS	RT	HF	1,114	China	(12)
	*NRXN2*	GWSS	Adaptation	HF	1,092	China	(100)

HSR, heat stress response; HT, heat tolerance; DS, drooling score; RT, rectal temperature; RR, respiration rate; TT, thermotolerance; TR, thermoregulation; GWSS, genome-wide signatures of selection; WGSS, whole genome detection of selection signature; ssGWAS, single step genome-wide association study; ssGBLUP, single step genomic best linear unbiased prediction; THA, Tharparkar; ONG, Ongole; HF, Holstein Friesian; AFR, Afrikaner; DRA, Drakensberger; NGI, Nguni; Eth and Bang, Ethiopia and Bangladesh; EASZ, East African Shorthorn Zebu. ^*^Genes replicated in different studies.

The identification of key candidate genes responsible for variation in thermoregulation could inevitably help to improve the efficiency of selective breeding, especially for traits with low heritability in dairy cattle breeding programs. In addition, investigation of the physiological systems regulated by genes involved in thermoregulation is an area that requires further study, as understanding the biological control will aid to prioritize candidate genes for genomic selection strategies (105). A considerable number of GWAS and transcriptome studies have been investigated and identified several candidate causal variants potentially linked with milk production and various physiological indicators of HS in dairy cattle (11, 12, 14, 15, 17–19, 24, 92–94, 96, 106, 107). Recent work has shown that the SNPs *rs8193046*, *rs43410971*, and *rs382039214*, within the *TLR4*, *GRM8*, and *SMAD3* genes, respectively, appear to be significantly associated with 305-day milk yield, RT and RR in Holstein dairy cows kept under heat-stress condition (94). Candidate genes *SPAG17*, *FAM107B*, *TSNARE1*, *RALYL* and *PHRF1* identified in Holstein cattle have been reported to be associated with RT, with *FAM107B* and *PHRF1* genes validated by functional analysis based on gene expression during thermal stress in a peripheral blood mononuclear cell model (12). The previous study by Luo et al. (12) evaluated physiological indicators of HS in Holstein dairy cattle and reported that *TSNARE1*, perhaps the potential gene, is significantly associated with RT. Luo et al. (14) also discovered seven major candidate genes (*PMAIP1*, *SBK1*, *TMEM33*, *GATB*, *CHORDC1*, *RTN4IP1* and *BTBD7*) associated with physiological indicators of HS (RT, RS and DS) in Holstein cattle by weighted single-step genome-wide analysis studies (WssGWAS). In the transcriptome study by Czech et al. (106), the *RAB39B* gene was found to be significantly associated with RT, DS and RS under HS conditions in dairy cattle. In addition, Diaz et al. (107) reported five genes: *E2F8*, *GATAD2B*, *BHLHE41*, *FBXO44*, and *RAB39B* which were significantly associated with HS in cattle. Dikmen et al. (16) reported two candidate genes (*ATPA1A* and *HSP70A*) associated with RT and RR during HS in lactating Holsteins. Previously, Dikmen et al. (24) also carried out GWAS for RT of lactating Holstein cows under HS conditions to find potential SNPs that could function as QTL for the same trait. They reported QTL markers that either contained or were in close proximity to specific functional genes such as *U1 spliceosomal RNA*, *NCAD*, *SNORA19*, *RFWD2*, *SCARNA3*, *SLCO1C1*, *PDE3A*, *KBTBD2*, *LSM5* and *GOT1*. Garner et al. (22) have already revealed *BDKRB1* and *SNORA19* as potential candidate genes associated with HS in dairy cows. A whole-genome association mapping study by Sigdel et al. (11) revealed that candidate genes *HSF1*, *MAPK8IP1*, and *CDKN1B* are directly involved in several cellular responses to HS in lactating dairy cows, for example activation of *HSP* (*PEX16*, *HSF1*, *EEF1D*, and *VPS28*), reduction of oxidative stress, modulation of apoptosis process (*MAPK81P1*, *CREB3L1*), DNA maintenance (*TONSL*), and thermotolerance (*CRY2*). The *UCN3* gene that is engaged in the genetic regulation of stress tolerance and oxidative stress in Holstein Friesian was described by (108).

A handful of studies have highlighted the impact of HS on the fatty acid profiles of milk from dairy cattle and identified genes potentially associated with response to HS. Recently, Bohlouli et al. (18) identified the candidate genes, *AMFR* for HS response of *PUFA*, *ADGRB1*, *DENND3*, *DUSP16*, *EFR3A*, *EMP1*, *ENSBTAG00000003838*, *EPS8*, *MGP*, *PIK3C2G*, *STYK1*, *TMEM71*, *GSG1*, *SMARCE1*, *CCDC57*, and *FASN* for HS response of *SFA*, *ENSBTAG00000048091*, *PAEP*, and *EPPK1* for HS response of UFA in dairy cattle and thus reported as a potential biomarker for heat tolerant animals. In another study looking at the candidate genes for coat color phenotypes of HS in cattle, Bahbahani et al. (98) indicated that *PMEL* is a strong candidate gene associated with eumelanin synthesis and thus could control coat color in cattle. In addition, the *ERBB3* and *MYO1A* genes have been proposed as the most likely candidate genes for coat color phenotypes in locally adapted tropical cattle breeds (31). It is indicated that the differences between Mongolian cattle (*Bos-taurus*) and Minnan cattle (*Bos-indicus*) resulting from *DVL2* mutations could alter the normal transcription and expression of the *DVL2* gene and also affect hair growth, allowing it to acclimatize hot-climate of southern China and cold climate of northern China (109).

The changes in cows’ response to HS can also be detected by the expression or activation of specific biological markers, particularly heat shock transcription factor (*HSF*), heat shock proteins (*HSP70*, *HSP90* and *HSP27*), *TLR2/4* and *IL2/6* (110). In particular, an evolutionarily conserved transcription factor known as heat shock transcription factor one (*HSF1*) binds the promoter regions of *HSPs* to control their stress-inducible synthesis in response to the environment (40). Heat shock proteins (*HSPs*) are highly conserved stress proteins that have been proposed as promising biomarkers of HS, of which *HSP70* has been shown to be upregulated in dairy cows’ mammary epithelial cells during HS to increase mammary gland thermotolerance (22). According to Liu et al. (111), heat-tolerant Chinese Holstein dairy cows had significantly higher plasma *HSP* (*HSP70* and *HSP90*) and cortisol levels compared with non-heat-tolerant dairy cows. Potential candidate genes such as *HSPA1B*, *HSPA1L*, *HSPA12A*, *GRXCR1*, *FKBP4*, and *IL-6* were reported to have a correlation with thermotolerance in tropically adapted dairy cattle breeds (102). The *HSPA9* and *HSPB9* genes, as well as two other members of the *DNAJ* family (*DNAJC8* and *DNAJC18*), have been linked to HS (97). Interestingly, highly significant causal mutation candidates in the *HSF1*gene for heat tolerance have been identified in Holstein cattle in Australia (23), and the United States (11), including *MGST1* (51). Transcriptome analysis by Liu et al. (111) identified the *OAS2*, *MX2*, *IFIT5*, and *TGFB2* genes associated with heat tolerance and involved in the immune effector process in dairy cows, with the *TGFB2* gene being part of the *MAPK* signaling pathway.

Specific biological pathways associated with nervous systems functions (interaction between neuroactive ligands and receptors, and glutamatergic synapses) and metabolism (citrate or Krebs cycle) are important factors in elucidating the mechanisms of heat tolerance in dairy cattle, warranting extensive follow-up of functional investigations (2). Cheruiyot et al. (93) attempted to conduct a GWAS study using several approaches, including conditional analyses for heat resistance employing a large sample size and genotype data set collected from dairy cows. They showed that the *ITPR1*, *ITPR2*, and *GRIA4* genes are related to the neuronal system and the *NPFFR2*, *CALCR* and *GHR* genes are related to neuroactive ligand-receptor interaction functions, which may be important for metabolic homeostasis in lactating dairy cows during thermal stress, with *GHR*, *NPFFR2* and *CALCR* providing the strongest evidence. In addition, Otto et al. (15) reported that the putative candidate genes, including *LIF*, *OSM*, *TXNRD2*, and *DGCR8*, undergo changes in biological processes in response to the effects of HS in crossbred dairy cows. On the contrary, the putative candidate genes *KIFC2*, *VPS13B*, *USP3*, and *SCD* have been reported to be involved in heat tolerance in lactating dairy cows, with the *SCD* gene encoding a fatty acid metabolism enzyme and possibly required for metabolic homeostasis during HS in mammals (93).

**Figure 3:**
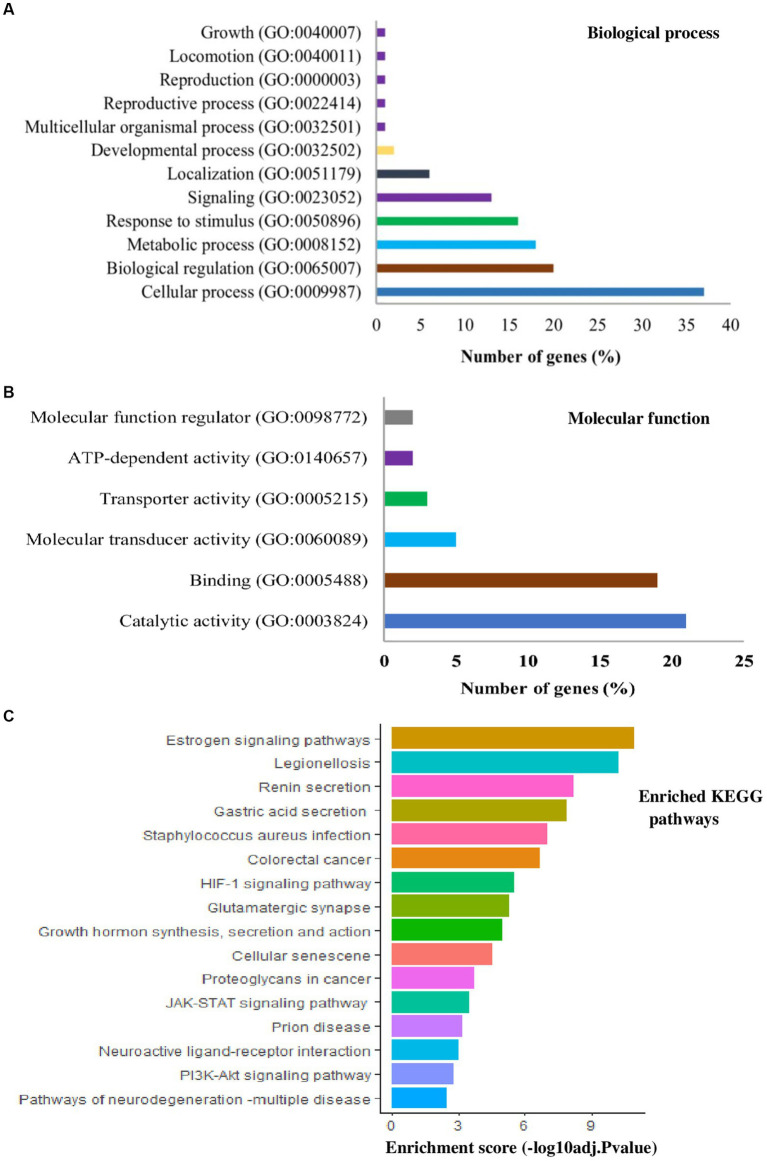
List of gene ontology (GO) terms and kyoto encyclopedia of gene and genomes (KEGG) pathways derived from the candidate gene list in Table 1 that were reported to be involved in various heat tolerance traits in dairy cattle population. Bars represent the number of genes for biological processes **(A)**, molecular functions **(B)**, and the enrichment score **(C)**.

Further, to see the specific biological functions, the lists of candidate genes for heat tolerance across different studies were subjected to GO functional annotation and KEGG pathway enrichment analysis. PANTHER^1^ and DVID^2^ data bases with cut-off point *p* < 0.05 and Benjamini-Hochberg false discovery rate (FDR) < 0.05 were used. In terms of biological processes, genes related to HS response as well as biological regulation, metabolic processes, or immune responses were found to be the most represented (Figure 3A), consistent with the proposed candidate genes and their ontology (40). In terms of molecular function, genes for catalytic activity and binding were the most represented (Figure 3B). Interestingly, estrogen signaling pathway is the top enriched pathway (*p* < 0.001), comprising of 10 candidate genes (*FKBP4, HSPA1L, ITPR1, ITPR2, KRT24, KRT25, KRT26, KRT27, KRT28, PFR*) involved in HS response in dairy cattle (Figure 3C). A total of 4 genes were enriched with legionellosis pathway (*HSPA1L, HSF1, 1 L6, TLR4*). The renin secretion pathway was enriched with 4 candidate genes (*AQP1, ITPR1, ITPR2, PDE3A*). It was also observed that the enrichment of genes in the neuroactive ligand-receptor interaction pathway (*CALCR*, *GRIA4*, *GRM8*, *GHR, MC5R, NPFFR2, PRLH, UCN3*), PI3K-Akt signaling pathway (*CDKN1B, GHR, IL6, MTOR, OSM, SGK1*, *TLR4*), HIF-1 signaling pathway (*CDKN1B, IL6, MTOR* and *TLR4*) and *JAK–STAT* signaling pathway (*LIF, GHR, IL6, MTOR* and *OSM*) with other pathways and stress responses (Figure 3C). In line with these pathways (Figure 3C), the previous studies found that the candidate genes for HS response enriched *HSF1*mediated heat shock response, estrogen signaling pathway, *HIF-1* signaling pathway, *PI3K-Akt* signaling pathway, *MAPK* singling pathway and immune response pathways in dairy cattle (93, 112, 113).

## 6. Signatures of selection for heat tolerance in dairy cattle

Selection pressure leaves distinct footprints in the areas of the genome that are exposed to selection called selection signature (25, 26, 114). When a derived allele or variant with fitness progress occurs in a population with positive selection, this results in neighboring related alleles being carried further alongside the selected variant (25). This indicates positions that are close to regions under selection are mostly affected by background selection and genetic hitchhiking (115). Thus, selection signature detection is used to identify these footprints/signs of selection (116), by which candidate genes related to a particular phenotype can be targeted. Understanding how selection acts on livestock genomes and detection of selection signature provides a better insight into the progress of artificial selection, which may especially benefit the optimization of breeding programs to improve animal resilience and other traits of economic importance (20, 25, 117). With the recent development and prevalent of high-throughput sequencing technologies along with various powerful statistical methods, there is an increased interest in genome-wide detection of selection signature, providing insights on the mechanisms of natural and/or artificial selection and uncovering candidate genomic regions under selection related to adaptation and climate resilience in cattle (20, 29–31, 100, 101, 118).

In cattle, genome-wide SNPs analysis has identified several candidate genome regions on BTA5, 6, 8, 23, and 26 under positive selection signature, containing interesting genes associated with heat tolerance in tropically adapted dairy cattle breeds (102). Moreover, the candidate region on BTA21 contains genes associated with heat tolerance suggested to be under selection in Shanghai Holstein cattle (100). A previous study focusing on the genomic signatures of divergent selection using high-density SNPs in taurine and Zebu cattle populations, already identified candidate genes associated with thermotolerance, including *HSP70*, *HSF1*, *CMPK1*, *NPM1*, and *GCN2* in Zebu cattle (31). Moreover, Freitas et al. (20) identified a positively selected candidate gene (*PLA2G2A*) underlying lipid metabolism in cattle in warm environments. Several other candidate variants and genes that may be responsible for differences in heat tolerance in particular environments and production systems have been discovered in potentially selected regions, including *ITGA9*, *ACAT2*, and *PLAC8* in dairy cattle (93). Saravanan et al. (102) documented genomic regions encoding candidate genes *HSPA1L*, *HSPA1B*, *DNAJB4*, *GRXCR1*, *OLA1*, *SP9*, and *HSPA12A* as selection signatures regulating heat tolerance in indigenous dairy cattle breeds. In addition, Liu et al. (100) identified a number of potentially and positively selected novel genes, such as *NDUFB3*, *RGS3*, *UBD*, *DIS3L2*, *NRXN2*, *PEX14*, *SPTLC2*, *AQP1*, and *PTPN9*, that are associated with adaptation to tropical humidity and harsh environmental conditions in Holstein cattle. Moreover, a previous finding in African cattle reported candidate genes *SLC9A4*, *PLCB1*, *FTO*, *ITPR2* (101), and *SOD1* (95) that have responded strongly to selection for heat tolerance. In addition, the *HSPA4* gene involved in protecting cells from heat damage and preventing protein denaturation showed selection success for heat tolerance in several cattle breeds in Africa (95) and China (109). Candidate genes studied, such as *AQP5*, *RAD50*, and *RETREG1*, have been shown to regulate acclimatization to heat in Russian cattle breeds (118).

## 7. Breeding strategies to increase heat resistance and productivity of dairy cattle

**Figure 4:**
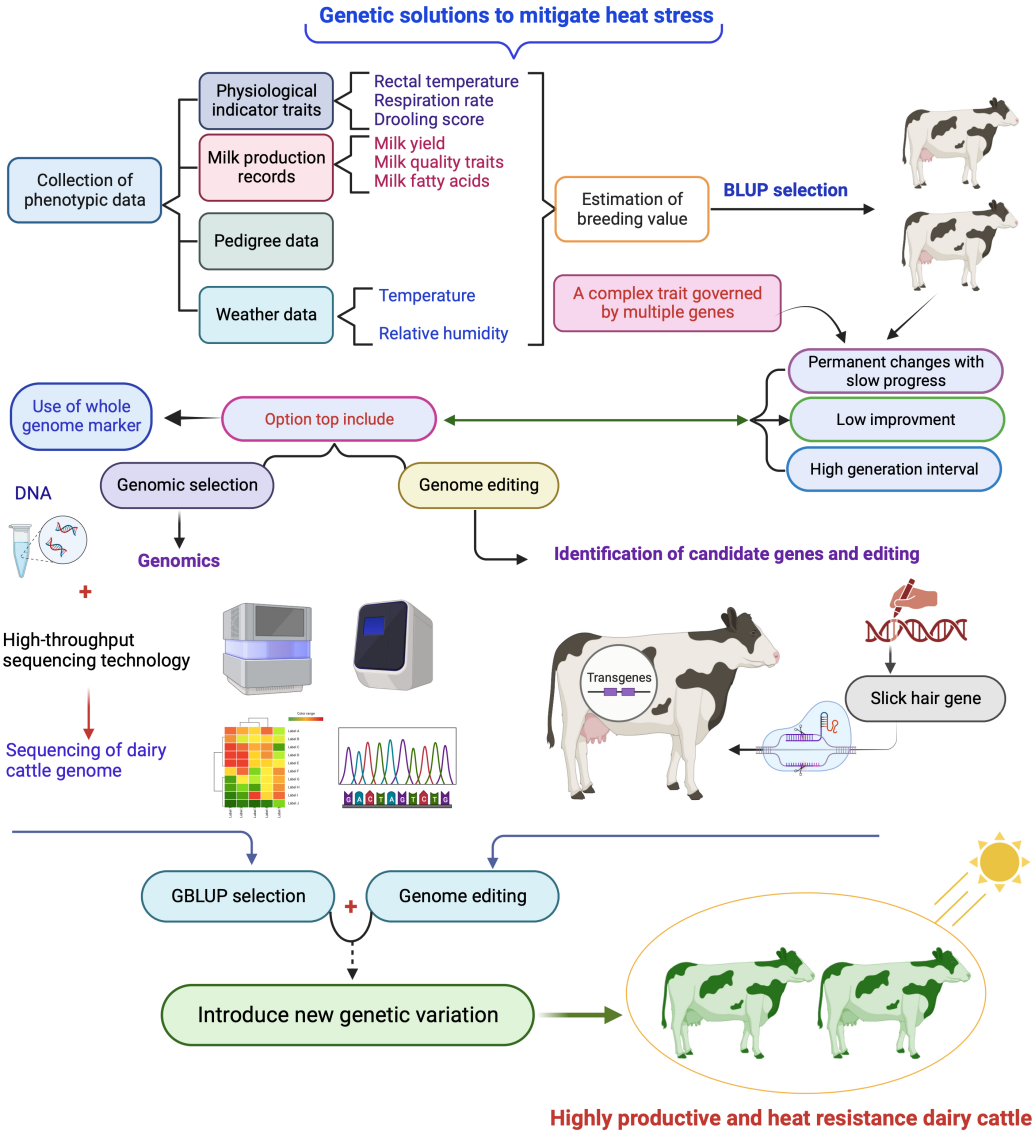
Brief overview of classical selection using phenotypic data (Best Linear Unbased Prediction (BLUP) selection) and genomic selection using genome-wide DNA markers (GBLUP) as well as genome editing to achieve optimal genetic progress for heat tolerance trait, while achieving genetic progress in milk production traits in dairy cattle. Created with BioRender.com.

Due to the increasing frequency and intensity of changes in temperature worldwide, identifying and developing dairy cattle resistant to HS is critical for breeding dairy herds that are better suited to future climatic challenges. In addition to known strategies to reduce the detrimental effects of HS, such as physical environmental modifications and improved feeding practices, the need for genetic progress, which includes selection (genetic and genomic) for heat tolerance with high milk yield is a current state of genetic research that could lead to a long-term solution to the problem (Figure 4). Also, these strategies might not be effective in pasture-based dairy production systems where dairy cows are exposed to solar radiation for much of their time while grazing (13). Several research attempts have been made to find breeding solutions for HS, which is already a feature of dairy cattle breeding programs in many regions. Breeding strategies for dairy cows through the genetic development of heat-tolerant breeds from genetic and genomic perspectives, targeted genome editing, and other options including epigenetic modifications are discussed in this section.

### 7.1. Breeding through crossing

The presence of genetic variation in thermoregulation in cattle breeds, i.e., the ability of some breeds to maintain body homeostasis, physiological functions, immune response and disease resistance under thermal stress, has opened up opportunities for research and identification of animals that can withstand severe environmental conditions (13, 62). Finding dairy cows that are genetically heat tolerant is another, perhaps more useful, improvement technique. The literature suggests that the *Bos indicus* breed can regulate its body temperature better than the *Bos-taurus* breed. Thus, *Bos-indicus* breeds are regularly crossed with *Bos-taurus* breeds to combine their ability to resist heat and parasites with the production qualities of temperate breeds (42). Although milk production is likely to decrease when a highly productive dairy cow, e.g., Holstein cow, is crossed with a Zebu, the magnitude of the decrease in milk production with increasing HS varies between the highly productive breeds and when crossed with Zebu. Evidence in the literature shows that HF cow with high genetic merit, as determined by their estimated breeding value for milk, show a greater decline in milk yield than their crossbred (HF x NZJ) counterparts (119), suggesting that highly productive dairy cattle are particularly sensitive to HS. Given that environmental HS is becomes a major challenge, especially for highly productive dairy cows, it impairs production and adaptation, and in extreme cases even leads to death. Therefore, it is inevitable to compensate for current and future economic losses through crossbreeding, considering the future benefits. In addition, crossbreeding or backcrossing between a locally adapted Zebu breed and a high milk yielding breed (e.g., HF) to capture heterosis for heat resistance and enhance milk production in crossbreds could be an alternative or even an additional solution, while optimizing productivity still requires further investigation (2). In this context, the use of crossbreds or synthetic breeds containing some proportion of *Bos-indicus* genetics, such as the Girolando breed in Brazil, which is the result of crossing Gir and Holstein animals, is an alternative for managing HS in tropical areas (105). However, cumulative genetic progress can be achieved in production by combining within-breed selection with reproductive technologies (such as artificial insemination and embryo transfer) to spread elite genomes more effectively (1). Nevertheless, selection within a breed may not be sufficient to meet the new challenges; perhaps the greatest benefit would come from selecting highly productive breeds and crossing them with adaptable local breeds, which could lead to rapid genetic changes (120). However, the breeding strategy to improve heat tolerance will be determined by the production system. In this case, production systems with limited resources would be more likely to benefit from crossing high-yielding breeds with local animals (40). Therefore, breeding by crossing local and selected breeds and selecting for improved productivity while monitoring heat tolerance appears to be the best approach to increase productivity in a production system that cannot provide heat protection, adequate nutritional conditions, and control of parasites and other pathogens (40).

Introgression strategies usually assume that one or more alleles in genes of interest or associated markers have been found in a donor population that are absent in the recipient population (121). Apart from the numerous candidate genes linked with the regulation of response to HS, the role of coat color attracts researchers’ attention. It is noteworthy that the slick-hair gene located on chromosome 20 originates from Senepol cattle on the Caribbean Island of St. Croix and is responsible for a smooth and short coat that confers thermotolerance, thus, associated with an enhanced ability to dissipate heat (51). It is interesting to note that Holsteins cows with the slick-hair phenotype have superior ability for thermoregulation than non-slick cows and experience a less decline in milk yield in a hot environment (122). According to the physiological parameters assessed (rectal temperature, respiration rate, skin temperature, and sweating rate), the SLICK1 allele was beneficial for conferring thermotolerance, as evidenced by lower rectal temperature in slick animals (123, 124). The slick-hair gene, which allows cows to better handle the heat by having short, smooth, and sometimes shiny hair, is used in breeding programs in many countries. Some countries, including Puerto Rico and the United States, have already started to include the slick variant in breeding programs for Holstein (40, 125), and carriers of the slick variant of Holstein bulls are already being marketed by artificial insemination companies. While the role of this variant in cold temperatures is still largely unclear, the slick mutation is also being targeted for gene editing, allowing faster introgression of the desired mutation with little to no background DNA from the donor breed (105). Remarkably, it is anticipated that genomic introgression from a highly adapted but low-production population into a highly productive but low adaptation population leads to accelerated genetic progress, perhaps, most successful when the adaptation trait is given less weighting than production traits in the selection index, potentially aiding to concurrent animal improvement (126).

### 7.2. Breeding by selection of heat-tolerant animals in high-output breeds

Inherent differences among animals in response to HS open the window for selection for heat-tolerant animals. Heat tolerance in dairy cows, as measured by RT, RR, DS, THI, or milk decline traits, is partially subject to genetic control with low (0.06) to moderate heritability (0.34) estimates (14, 50, 89), suggesting that a response to selection can be expected for these traits in the dairy cattle population. While physiological measures such as changes in core body temperature in animals are often used to partially capture genetic effects for heat tolerance in cattle, these traits are unfavorably correlated with milk production (2, 77, 83, 87). Given these findings, Brito et al. (86) proposed that it is of paramount importance for the dairy industry to consider traits that capture heat dissipation efficiency, so that selection of high-ranked heat-tolerant animals with superior genetic merit to remove metabolic heat from the core body into the environment allows for more sustainable genetic progress towards milk production. The combination of several heat tolerance measures is now becoming a common feature in the dairy industry to maximize dairy cattle productivity. For example, in the Australian dairy industry, sires have already been selected based on genomic breeding value for their tolerance to HS in relation to changes in milk, fat, and protein yield per unit increase of THI (50). Although physiological indicators such as changes in core body temperature are often seen as the gold standard for heat tolerance in cattle (40), their use in large-scale genomic evaluation is still limited because it is too costly and labor-intensive to take measurements of body temperature on thousands of animals that would be required for genomic evaluation (2).

Dairy cattle breeding programs for heat tolerance could be accelerated by GS, which uses genome-wide DNA markers that predict tolerance to HS and is more efficient and appropriate than traditional genetic evaluation using pedigree. These DNA markers are often SNPs used to predict genomic breeding values. In addition, GS can be a cost-effective method to apply an efficient breeding approach, even in low and middle-income countries (127). Nevertheless, heat tolerance breeding values have an unfavorable genetic association with milk production traits (49), so selection for higher milk production may have inadvertently also selection for dairy cows with less heat tolerance, implying that they are regulated by the same genes. For example, a genetic selection program to increase milk production in Holsteins (128) and Gyr breeds in the tropics (90) negatively affects the animals’ ability to cope with HS. However, experience in GS of dairy cows shows that it is possible to improve multiple traits of economic importance simultaneously, even if there is genetic antagonism between them (129). For example, Australia is currently pioneering the use of genomics to improve heat tolerance in dairy cattle (49, 50) and is providing stand-alone genomic breeding values for this trait to help farmers choose heat-resilient animals (2). To do this, farmers use a two-step approach to first filter bulls based on a balanced performance index, that encompasses a wide range of traits, such as production, health, fertility, type, and feed efficiency (130) and then on heat tolerance (2). Alternatively, it is possible to select animals with high heat tolerance breeding values within highly productive breeds and incorporate resilience indicators, for example, the milk yield in heat-tolerant cows and recovery period following heat challenge compared to heat-susceptible cows, into breeding objectives are a critical part of the strategy for breeding cattle adapted to warmer environments (2). In a nutshell, in dairy cattle, it is important to identify genetics that confers resistance to HS while maintaining a desired level of production.

### 7.3. Breeding for heat resistance by programmable CRISPR-Cas9-based nucleases

Under genome editing, genetic improvement would no longer require that the variants exist in the breed of interest; rather, beneficial mutations could be transferred between populations and species or even designed at will (1). The development of programmable nucleases, including Fok1-based zinc-finger nucleases (ZFNs), transcription activator-like effector nucleases (TALENs) and more recently CRISPR-Cas9-based nucleases, have fundamentally changed the picture (131). Tyagi et al. (132) demonstrated that genome editing through the CRISPR-Cas9 system could enable dairy cows to adapt more effectively to environmental conditions or specific production systems, which could improve production, reproduction, disease resistance and animal welfare in herds. The CRISPR-Cas9 system made it possible to maintain or even accelerate the rate of genetic gain already achieved through conventional breeding programs by introducing desired alleles, such as those related to heat tolerance or disease resistance, into dairy breeds (133). It is worth mentioning that the gRNA/Cas9-mediated precise introgression of the naturally occurring mutation *p. Leu18del* in the pre-melanosomal protein 17 (*PMEL*) gene, known from Galloway and Highland cattle, in Holstein-Friesian cattle, confirmed the causative status of a coat color phenotype mutation to a changed environmental condition in cattle (134). In addition, a deletion of a leucine codon in the signal peptide (*p. Leu18del*) of *PMEL* that segregates in Highland, Galloway, and Tuli cattle has been proposed as a causative mutation for coat color effects (135). Therefore, the effects on coat color make it an excellent candidate for introgression into Holstein-Friesian dairy cows to reduce radiative heat gain and improve overall heat tolerance. Another causal variant useful for introgression is a deletion in the promoter regions of the heat shock protein (*HSPA1*) gene, which provides cellular protection from high temperatures under HS conditions (125). Genomic introgression could thus be effectively used to improve the adaptation of high-yielding animals (126), leading to low inbreeding rates. However, there are ethical issues related to this approach that argue both for and against genome editing in various animal species. It is argued that conducting genome editing experiments would itself cause suffering of animals. In addition, genome editing could lead to off-target mutations or unintended effects, which could negatively impact animal health. On the other hand, genome editing could be used to reduce animal suffering by making dairy animals more heat-resistant given future environmental consequences. At the same time, genome editing could be done in other ways, and it is possible to make the public to consider this approach in dairy farming less controversial than others using gene drive designs. One-way is to involve members of the community breeding program.

### 7.4. Breeding by epigenetic modifications and thermal imprinting of the genome

Genomic variations can explain a portion of the various phenotypic traits, including the stress response, and the remaining part is probably embedded in the epigenome and its dynamic interplay with environmental stimuli. Epigenetics is defined as the study of heritable molecular changes that regulate gene expression and other genomic functions, leading to phenotypic variations without altering the underlying DNA sequence (136, 137). Given this, epigenetics shows that not all genetic information is contained in the DNA sequence, but also in some modifications that take place throughout the epigenome (138). The epigenome modifications, which include DNA methylation (DNAm), histone modifications, non-coding RNAs (ncRNA) and chromatin remodeling respond to environmental cues to influence the expression of genes and specific phenotypes, suggest the influence of epigenome variations on environmental adaptation and other economic important traits in livestock (138–140). This necessitates the identification of specific epigenetic marks that are triggered by specific environmental stresses in the epigenome of cattle. These environmental stress-sensitive epigenetic marks or regions could then be used as a molecular biomarker to assess HS in dairy cows and may hold promise for genomic selection to improve heat-tolerance in dairy herds. In this case, genotypes for high epigenetic potential regardless of changing environments could be used for the selection of dairy cows that enable them to respond to a stressful environment. Thus, it is imperative that attention should be given to understanding the influence of epigenome alterations and application in dairy cattle breeding, which is vital for the effective exploitation of epigenetic information for sustainable heat tolerance trait improvement in dairy cattle. Thanks to the recent advancements in high-throughput sequencing technology, including bisulfite sequencing-based technologies (BS-seq) and chromatin immune precipitation sequencing (CHIP-seq), epigenetic markers on a genome-wide basis in cattle can be quantified.

DNA methylation is thus far the most stable and extensively studied epigenetic modification in most mammalian genomes (140), which is best known for its function in genomic imprinting and X chromosome inactivation and is crucial for transcriptional regulation throughout the genome (141). Heat stress alters DNAm of promoter regions and alters gene expression through other epigenetic modifications, including histone modifications and microRNAs (miRNAs), which in turn contribute to variation in response to HS (142). On the other hand, Skibiel et al. (143) noted that heat-induced changes in DNAm may not play a significant regulatory role in gene expression; rather, HS may alter gene expression through histone modifications and miRNAs. Although studies on the mechanisms by which epigenetic regulation influences the response to HS in cattle are still largely unclear, Livernois et al. (142) performed genome-wide DNAm from blood samples of high and low-immune responder heat-stressed Holstein dairy cows. The DNAm analysis from high immune responder cows revealed that differential DNAm of promoter genes is associated with stress response and apoptosis prevention form. Whereas in low immune responder Holstein cows, HS affected promoter methylation of genes associated with cell proliferation and histone deacetylases. Notably, susceptibility and dynamic epigenetic changes are shown in response to drastic temperature fluctuations in bovine pre-implantation embryos (144). Garner et al. (22) evaluated the seasonal HS effect on transcriptomic profiles and global DNAm of bovine oocytes. The study revealed a substantial number of genes and pathways regulated by seasonal HS. However, no differences were found in the global levels of DNAm and DNA hydroxy-methylation of oocytes collected in the different seasons. Heat stress effect in lactating cows alters gene expression and influences the global DNAm, perhaps affecting the mechanisms of postnatal HS response, which may contribute to future performance in calves (143, 144).

Histone modification is another important epigenetic mechanism from activation to epigenetic regulation of transcription of HS-related genes (e.g., *HSPs*) during embryonic development in response to HS (144–146). This would lead the embryo resilience to HS in later life, which may impact dairy cattle productivity and adaptation. In addition, HS upsets the epigenome of dairy cattle offspring by altering gene expression, which subsequently affects embryo chromatin and induces the aggregation of histone H3 lysine 9 trimethylation, histone H3K9 hypoacetylation and heterochromatin protein 1 (144, 147), possibly affecting the future performance of offspring into adulthood (143).

Genomic imprinting is another form of epigenetic regulation, in which alterations in gene expression do not require changes in the underlying DNA sequences, but rather the expression of a gene depends on its parental origin (148). This can profoundly influence phenotypic variation in adaptation and production traits in cattle. Epigenetic regulation of gene expression and thermal imprinting of the genome could also be an efficient method to improve thermal tolerance. Further epigenetic control is provided by miRNAs, which have emerged as factors in transcriptional regulation and HS memory and are involved in HS adaptation by acting as post-transcriptional regulators (149). Nevertheless, many important aspects of imprinting and epigenetic control remain to be elucidated. Several previous reports present evidence of imprinted QTL, which encompasses the bovine imprinted genes, *GNAS* and *PEG3* influencing growth and carcass traits in cattle (150) and the callipyge locus on *OAR18* influencing body weight, muscle and fat depth measurements in Texel sheep (151). Imprinted gene expression and genetic variation at imprinted loci may have significant effects on dairy cattle HS response. For example, the imprinted genes *DLK1* and *DIO3* expressed at postnatal stages are associated with non-fresh thermogenesis in brown adipose tissue, which is essential to prevent hypothermia (152), and other imprinted genes such as *Gnas*, *Gnasxl*, *Ndn*, and *Dio3* are involved in brown adipose tissue metabolism (153). Therefore, uncovering genome-wide imprinted genes associated with HS and production traits in cattle and taking them into account in genomic selection could help to achieve rapid genetic progress in improving these traits in dairy cattle.

In a nutshell, we illustrate the importance of epigenetic variations and more particularly DNAm as a useful biomarker for environmental stress, as DNAm is sensitive to the environment and is involved in organisms’ plastic and adaptive response to the changing environment (154). The current dairy breeding industry can benefit from the use of epigenetic biomarkers for dairy breeding programs. Since the genetic data currently used for livestock breeding can only explain a portion of the phenotypic variation or trait heritability, the addition of epigenetic biomarkers to genetic data could help improve the prediction accuracy of breeding values. If epigenetic variation due to imprinted genes is high, it can be used for selection of male and female lines. In addition, it may be useful for mating design to consider the imprinting status of the most favorable epigenetic status to complement the breeding value.

## 8. Conclusions and prospects for the future

In this review, we provide a comprehensive overview of the genomic regions and candidate genes associated with heat tolerance that can expand our knowledge to the genetic mechanisms of HS, which could open new avenues for mitigating the impacts of global warming on dairy cows. Many dairy farmers use a variety of strategies to keep their cows cool, such as shade, ventilation, cooling with water, drinking water, cooling in the barn, fans, sprinklers, etc. However, these strategies are quite costly to be of practical use in extensive dairy production systems under smallholder conditions, especially in hot, less developed and agriculture-dependent countries. As the effect of global warming intensifies, even locally adapted dairy breeds could be affected, and future dairy farmers will suffer the greatest economic losses. This will cause farmers to switch from dairy production to other sectors, such as beef production, as dairy cows are more affected by HS than beef cattle. Therefore, breeding dairy cows with greater heat tolerance ability, perhaps through genetic means, and the associated support in these vulnerable regions for food security must be addressed. While identifying heat-tolerant dairy cattle is challenging due to the complex phenomenon of HS and the antagonism between heat tolerance and production traits, the use of genome-wide information could be a way to unravel the genetic mechanisms of heat tolerance in dairy cows, that can be accustomed to the selection programs. Progress has been made in using genomic information to identify candidate genes and causative variants associated with heat tolerance and milk production traits in dairy cattle, that can be used for marker-assisted selection, genomic selection and gene editing programs. The literature shows that heat tolerance is a complex trait influenced by many genes in the genome, with the specific genes *HSF1*, *SPAG17*, *DNAJC8*, *HSPA9* and *DNAJC18* appears to have been reported in more than one independent studies. Further studies of these genes could help to understand the genetic mechanisms of adaptation to HS, which may help to simultaneously improve heat tolerance and production traits in dairy cattle. In addition, optimal breeding strategies for the genetic development of heat-tolerant dairy cows provide long-term solutions to HS effects, that are essential for addressing the dual challenge of increasing dairy production to feed the increasing human population, while addressing the impacts of global warming. Overall, this review could serve as a valuable resource material for the dairy breeding industry aimed at increasing heat tolerance, while maintaining milk production traits.

## Author contributions

DW conceived, conducted the literature search, performed visualization, and wrote the first draft. JH, GM, and BS provided critical appraisal and suggestions. MA involved in conceptualization, writing – review and editing. All authors contributed to the evaluation, editing, and approval of the final version of the manuscript.

## Conflict of interest

The authors declare that the research was conducted in the absence of any commercial or financial relationships that could be construed as a potential conflict of interest.

## Publisher’s note

All claims expressed in this article are solely those of the authors and do not necessarily represent those of their affiliated organizations, or those of the publisher, the editors and the reviewers. Any product that may be evaluated in this article, or claim that may be made by its manufacturer, is not guaranteed or endorsed by the publisher.

## Footnotes

^1^ https://doi.org/10.1093/nar/gky1038

^2^ https://David.ncifcrf.gov/
